# Urinary N-acetyl-D-glucosaminidase can predict bleeding after a percutaneous kidney biopsy

**DOI:** 10.1186/s12882-024-03658-z

**Published:** 2024-07-22

**Authors:** Hiroyasu Goto, Yota Kobayashi, Hiroki Sato, Tsugumi Fukunaga, Keiko Tanoue, Aoi Yamashiro, Hidehito Matsubara, Naoki Oshima

**Affiliations:** Department of Nephrology and Endocrinology, National Defense Medical College, 3-2 Namiki, Tokorozawa, Saitama 359-8513 Japan

**Keywords:** Percutaneous kidney biopsy, N-Acetyl-β-D-glucosaminidase (NAG), Bleeding risk, Arteriosclerosis

## Abstract

**Background:**

A percutaneous kidney biopsy (PKB) allows nephrologists to make informed decisions for treating various kidney diseases; however, the risk of bleeding complications should be considered, given the vascularity of the kidney. Many studies have reported risk factors for bleeding events after a PKB. However, while urinary N-acetyl-β-D-glucosaminidase (NAG) is a useful biomarker of kidney disease severity, little is known about whether or not urinary NAG is related to the bleeding risk.

**Methods:**

Medical records of patients who underwent a PKB at the National Defense Medical College Hospital between October 2018 and October 2023 were retrospectively studied. Hemoglobin (Hb) loss ≥ 1 g/dL was defined as a bleeding event.

**Results:**

Of the 213 patients, 110 (51.6%) were men, and the median age was 56 years old (interquartile range 40–71). The most frequent diagnosis on a PKB was IgA nephropathy (*N* = 72; 34.0%). Fifty-four patients (25.3%) experienced Hb loss ≥ 1 g/dL after a PKB, and urinary NAG/Cr levels before the biopsy were able to predict a bleeding event, with an area under the receiver operating characteristic curve of 0.65 (*p* = 0.005). Using the optimal cutoff value of 35 U/gCr, urinary NAG/Cr was found to be an independent risk factor by multiple logistic regression analysis (odds ratio 3.21, 95% confidence interval 1.42–7.27, *p* = 0.005). Even after adjusting for previously-reported risk factors, the elevated urinary NAG/Cr ratio remained a statistically significant variable. Compared with the pathological findings, only the severity of multilayered elastic laminae of the small muscular artery was associated with both urinary NAG/Cr levels (*p* = 0.008) and bleeding events (*p* = 0.03).

**Conclusion:**

Urinary NAG successfully predicted not only the severity of kidney disorders but also bleeding events after a PKB. Arteriosclerosis in the kidneys may be the mechanism underlying these increased bleeding events.

**Supplementary Information:**

The online version contains supplementary material available at 10.1186/s12882-024-03658-z.

## Introduction

A percutaneous kidney biopsy (PKB) is the gold-standard procedure for diagnosing kidney disorders, such as glomerulonephritis and nephrotic syndrome. Since the 1950s, when it was first reported, biopsy techniques and equipment have advanced substantially, and the diagnostic ability and complication risk have been improved [1]. However, the risk of bleeding after a biopsy is still present, although the incidence rates of blood transfusion and intervention after a PKB are < 1%[1], whereas the rate of minor complications, including hemoglobin (Hb) loss ≥ 1 g/dL, has been reported to be relatively high at 10–20% [2, 3].

Several studies have investigated the risk factors associated with bleeding complications after a PKB. An older age [4], female sex [4, 5], high serum creatinine level [5-7], acute kidney injury (AKI)[5, 8], low platelet count [8], low Hb level [2, 6, 8], prolonged active partial thromboplastin time (APTT) [2, 4], and frequent needle passes [7, 9] have been reported as risk factors for bleeding events due to a PKB. However, to our knowledge, no study has focused on the association between urinary biomarkers and the risk of bleeding after a PKB.

Urinary biomarkers, such as urinary N-acetyl-β-D-glucosaminidase (NAG) and β2 microglobulin (β2MG), are rapid and robust assays that are widely used to detect proximal tubular damage. In particular, because NAG is a large molecule and it cannot pass through glomerular filtration, the level of urinary NAG is thought to reflect the kidney status [10]. Thus, we focused on urinary NAG in this study. Since 1969, when urinary NAG was first described as a kidney injury marker [11], a large number of studies have confirmed the usefulness of urinary NAG in detecting kidney injury. High levels of urinary NAG appear to be associated with severe glomerulonephritis [12] and lupus nephritis [13], poor steroid sensitivity of nephrotic syndrome [14], and an advanced stage of diabetic kidney disease (DKD) [15], which are generally considered to carry a high risk of bleeding after a PKB. Therefore, we hypothesized that urinary NAG could predict bleeding risk after a PKB, reflecting the status of kidney pathology in various kidney disorders.

We therefore studied whether urinary NAG levels could predict bleeding risk after a PKB. We also investigated the pathological findings of PKBs underlying both bleeding complications and increased urinary NAG levels.

## Materials and methods

### Study design

**Fig. 1:**
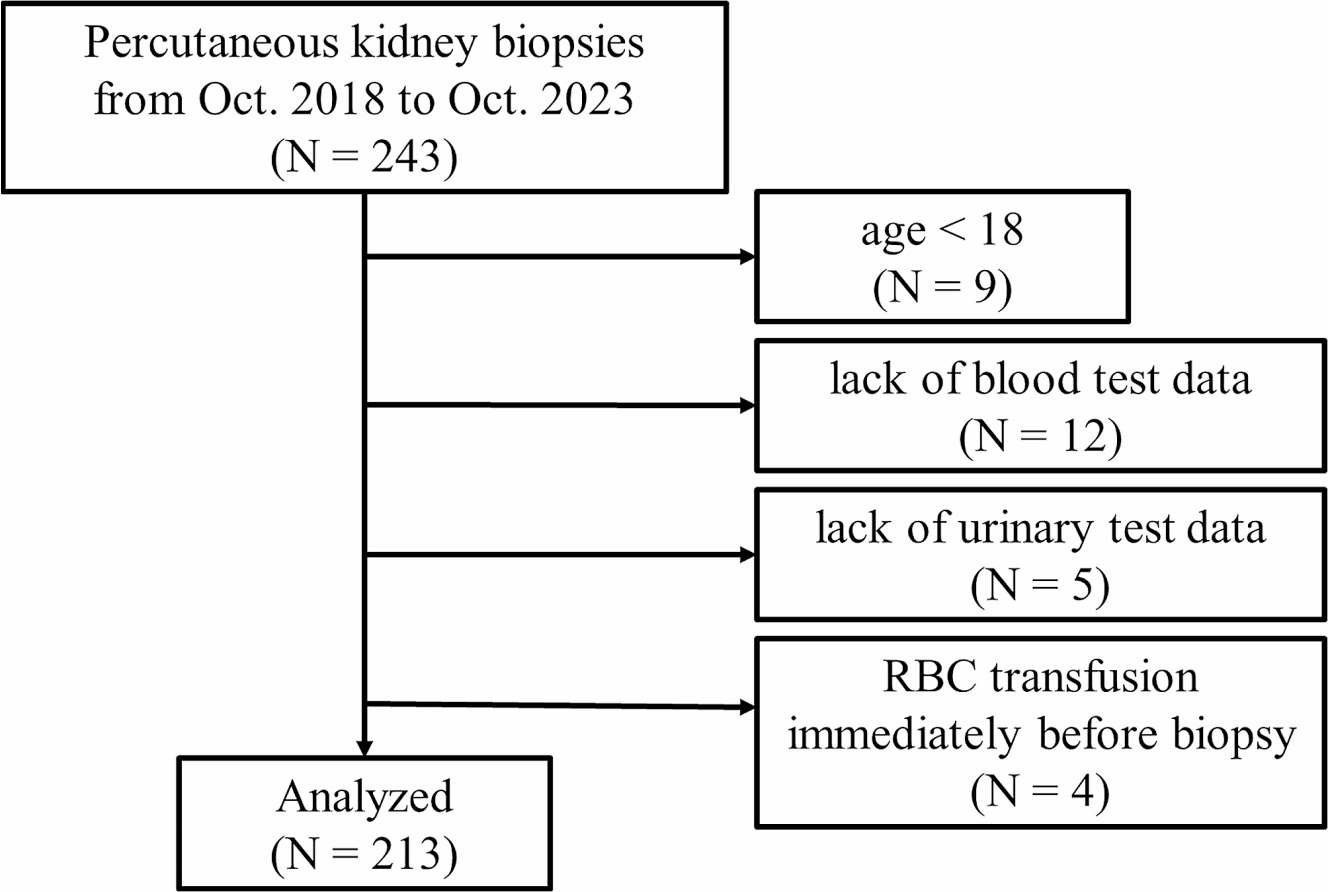
The flowchart of patient selection in this study. Data on patients who received percutaneous kidney biopsies at our institute from October 1, 2018, to October 1, 2023, were collected for this case-control study. Patients who were under 18 years old or lacked blood or urine test data were excluded. We also excluded patients who received blood transfusions immediately before PKB (*n* = 4). Finally, data from 213 patients were analyzed

We conducted a retrospective case-control study that focused on the association between urinary NAG levels and bleeding events after a PKB. We collected data from 243 patients who underwent a percutaneous native kidney biopsy in the Department of Nephrology at the National Defense Medical College Hospital between October 1, 2018, and October 1, 2023. At our institute, we perform approximately 40 to 50 PKBs annually. Patients under 18 years old (*n* = 9) were excluded, as were patients with missing essential data (listed in Table 1) on blood (*n* = 12) or urine (*n* = 5) tests. We also excluded patients who received blood transfusions immediately before PKB (*n* = 4) because the Hb levels after PKB could be influenced by the blood transfusion. The data from 213 patients were ultimately analyzed (Fig. 1).

**Table 1:** Patient characteristics

	*N* = 213
Age	56 (40, 71)
Sex-Male, N (%)	110 (51.6)
Clinical diagnosis before biopsy, N (%)	
Acute nephritic syndrome	19 (8.9)
Chronic nephritic syndrome	63 (29.6)
Nephrotic syndrome	51 (23.9)
Isolated hematuria	26 (12.2)
Isolated proteinuria	34 (16.0)
Acute kidney injury	13 (6.1)
Chronic kidney diseases	7 (3.2)
sBP (mmHg)	136 (122, 153)
dBP (mmHg)	80 (70, 90)
BMI	22.3 (20.2, 25.0)
Serum Alb (mg/dL)	3.7 (2.6, 4.1)
Serum Cr (mg/dL)	1.06 (0.79, 1.66)
eGFR (mL/min/1.73m^2^)	53.6 (33.7, 72.8)
Hb (g/dL)	12.7 (11.1, 14.2)
Plt (×10^4^/µL)	24.2 (19.6, 33.4)
PT-INR	0.97 (0.94, 1.01)
APTT (sec)	28.4 (26.7, 30.5)
Upro/Ucr (g/gCr)	1.52 (0.51, 4.21)
Diabetes, N (%)	28 (13.1)
Hypertension, N (%)	102 (47.4)
Dyslipidemia, N (%)	60 (28.2)
Heart disease, N (%)	16 (7.5)
Current smoker, N (%)	90 (42.3)
Needle passes	3 (3, 4)

Data are presented as the median and interquartile range or number (%). Pairwise group comparisons were performed using the Mann-Whitney U test. Categorical data were analyzed using chi-squared test

sBP, systolic blood pressure; dBP, diastolic blood pressure; BMI, body mass index; Alb, albumin; Cr, creatinine; eGFR, estimated glomerular filtration rate; Hb, hemoglobin; Plt, platelet; PT-INR, prothrombin time international normalized ratio; APTT, activated partial thromboplastin time; Upro, urinary protein

This study was approved by the Research Ethics Committee of the National Defense Medical College (Clinical Trial Number, 4914) and conducted in compliance with the Declaration of Helsinki.

### Percutaneous kidney biopsy (PKB) *procedure*

The indications and contraindications for PKB at our institution are summarized in Supplemental Table 1, which was modified from previous reports [1, 16]. A well-trained nephrologist who practiced for 5–8 years performed a PKB under the supervision of an educator of the Japanese Society of Nephrology. Following local anesthesia, a PKB was performed under ultrasound guidance using a 16-gauge automated spring-loaded gun. Most specimens were obtained from the lower pole of the left kidney (95.9%). The number of needle passes was determined when the appropriate specimens were observed using a stereomicroscope. Patients with uncontrolled hypertension (systolic blood pressure > 160 mmHg) were continuously administered intravenous nicardipine during the biopsy. After obtaining appropriate specimens, the puncture site was pressed firmly for approximately 15 min, and then the patients were kept in the supine position until the next morning. The next morning, ultrasound images and complete blood counts were performed to identify bleeding complications.

### Data collection

Patient age, sex, comorbidities, medications, and blood and urinary test data were collected from medical records. Information on pathological findings was collected from reports written by trained pathologists. Blood and urinary samples were analyzed at the Laboratory of the National Defense Medical College Hospital. The estimated glomerular filtration rate (eGFR) was calculated using the Japanese equation of the eGFR for serum creatinine (Cr) [17]. Urinary protein, NAG, and β2MG levels were normalized to urinary Cr levels. The reference range of active partial thromboplastin time (APTT) in our institute was 24 to < 34 s.

### Measurement of Hb levels and definition of bleeding event

Patients had blood samples collected while in a sitting position to avoid hemoconcentration, as fluids pool in the lower extremities. Venous blood samples were collected in Na_2_EDTA-coated tubes and the complete blood count (CBC), including Hb, was analyzed using an XR9000 analyzer (Sysmex Corp., Kobe, Japan). Hb loss was calculated using the most recent Hb level before the biopsy and the Hb level on the day after the biopsy. Bleeding events were defined as an Hb loss of ≥ 1 g/dL, as previously reported [2, 18].

### Determination of predictors of bleeding complications

Significant independent predictors of the post-biopsy bleeding events were determined using a multiple logistic regression analysis. Baseline factors that showed statistical significance in a univariate analysis were initially included as confounding factors. However, this method was previously reported to wrongly reject potentially important variables [19]. Therefore, we generated another multivariable logistic regression model using previously reported risk factors as confounding variables. In this analysis, we selected confounding variables from a recent large cohort study [5]. This study established a risk score for post-biopsy bleeding, and we selected anemia, vasculitis, and AKI, which each had a score of ≥ 4. Thrombotic microangiopathy was not included as a confounding variable because no patients had thrombotic microangiopathy in the current study.

### Statistical analyses

Data are presented as medians and interquartile ranges (IQRs) for continuous data and as numbers and percentages for categorical data. Pairwise group comparisons were performed using the Mann-Whitney U test. Receiver operating characteristic (ROC) curves for predicting bleeding complications were drawn, and the area under the curve (AUC) and optimal cutoff values were calculated. Categorical data were analyzed using the chi-squared test or Fisher’s exact test. Statistical significance was set at *P* < 0.05. All statistical analyses were performed using the JMP software program (version 15; SAS Institute, Cary, NC, USA).

## Results

### Patient characteristics

Of the 243 patients who received a PKB between October 1, 2018, and October 1, 2023, at our institute, 30 were excluded based on the exclusion criteria, and 213 were ultimately analyzed. Patient characteristics are shown in Table 1. Of the 213 patients, 110 (51.6%) were men, 28 (13.1%) had diabetes, and 102 (47.4%) had hypertension. The median (IQR) values for the age, body mass index (BMI), and eGFR were 56 (40–71) years old, 22.3 (20.2–25.0) kg/m^2^, and 53.6 (33.7–72.8) mL/min/1.73m^2^, respectively. The median (IQR) blood pressure at the beginning of the biopsy was 136/80 (122–153/70–90) mmHg. The needles were used for a median (IQR) of 3 (3–4) passes.

The histopathological diagnoses of the PKBs are shown in Table 2. The most frequent histopathological diagnosis was immunoglobulin A (IgA) nephropathy (*n* = 72, 34.0%), followed by membranous nephropathy (*n* = 17, 8.0%) and tubulointerstitial nephritis (*n* = 14, 6.6%). Biopsy specimens from 4 patients were inadequate for a histopathological diagnosis due to too few glomeruli (< 7 glomeruli [20]).

**Table 2:** Histopathological diagnoses of patients

	*N* = 213
IgA nephropathy	72 (34.0)
Membranous nephropathy	17 (8.0)
Tubulointerstitial nephritis	14 (6.6)
ANCA-associated nephritis	14 (6.6)
Lupus nephritis	13 (6.1)
Minimal change disease	10 (4.7)
Focal segmental glomerulosclerosis	10 (4.7)
Diabetic kidney disease	9 (4.2)
IgA vasculitis	4 (1.9)
Hypertension and ischemic renal injury	4 (1.9)
Membranoproliferative glomerulonephritis	3 (1.4)
Renal amyloidosis	2 (0.9)
IgG4-related disease	2 (0.9)
Light chain deposition disease	2 (0.9)
Others	9 (4.2)
Undefined nephropathy	24 (11.1)
Inadequate material	4 (1.9)

Data are presented as the number (%)

IgA, immunoglobulin A; ANCA, anti-neutrophil cytoplasmic antibody; IgG, immunoglobulin G

### Incidence rate of Hb loss ≥ 1 g/dL

Fifty-four (25.3%) patients experienced Hb loss ≥ 1 g/dL due to a PKB. Only 2 (0.9%) patients received blood transfusions after the biopsy, with an Hb loss of 2.2 and 3.4 g/dL reported. The patient with 3.4 g/dL Hb loss underwent a computed tomography (CT) scan, which showed a large hematoma (estimated volume of 155 mL). Other patients were found to have no or mild hematoma on routine ultrasound the day after PKB. Thirteen patients (6.1%) had macroscopic hematuria after PKB, which improved with supportive care, which was continued until the next morning. No post-biopsy interventional radiology or nephrectomy was required to control hemorrhaging. None of the patients died due to a PKB in this case-control study.

**Fig. 2:**
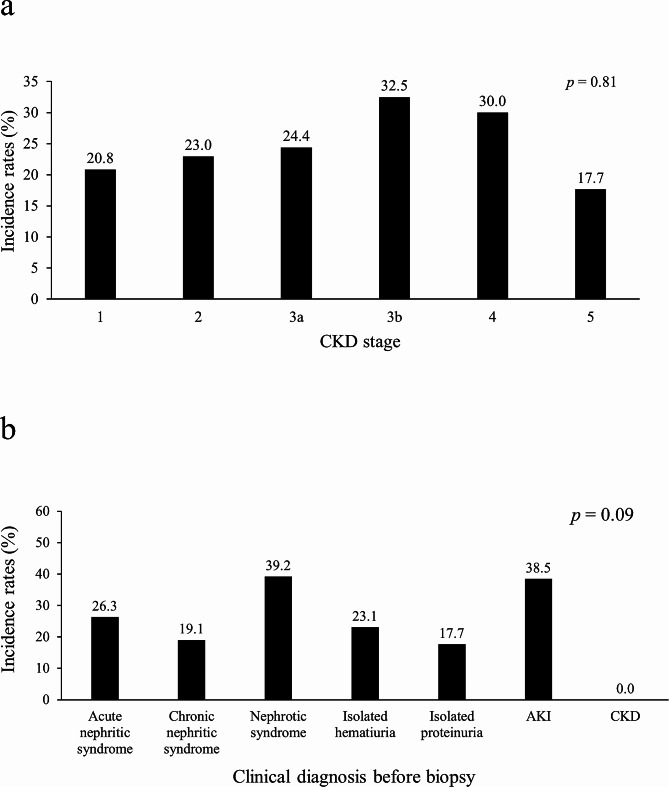
Incidence rate of hemoglobin (Hb) loss ≥ 1 g/dL after percutaneous kidney biopsy. The comparison of the incidence rate of Hb loss ≥ 1 g/dL among chronic kidney disease (CKD) stages (**a**) and clinical diagnoses before a biopsy (**b**). There were no differences in rates among CKD stages or clinical diagnoses. The numbers on the top of the bar graph represent the incidence rate as a percentage. Data were analyzed using Fisher’s exact test. AKI: acute kidney injury, CKD: chronic kidney disease

The incidence rate of Hb loss ≥ 1 g/dL did not markedly differ among the stages of chronic kidney disease (*p* = 0.81, Fig. 2a). Patients who were clinically diagnosed with nephrotic syndrome and acute kidney injury (AKI) before the biopsy seemed to have a high incidence rate of Hb loss ≥ 1 g/dL, although there were no statistically significant differences among clinical diagnoses before the biopsy (*p* = 0.09, Fig. 2b).

### Predictive ability of urinary NAG for incidence of Hb loss ≥ 1 g/dL

**Fig. 3:**
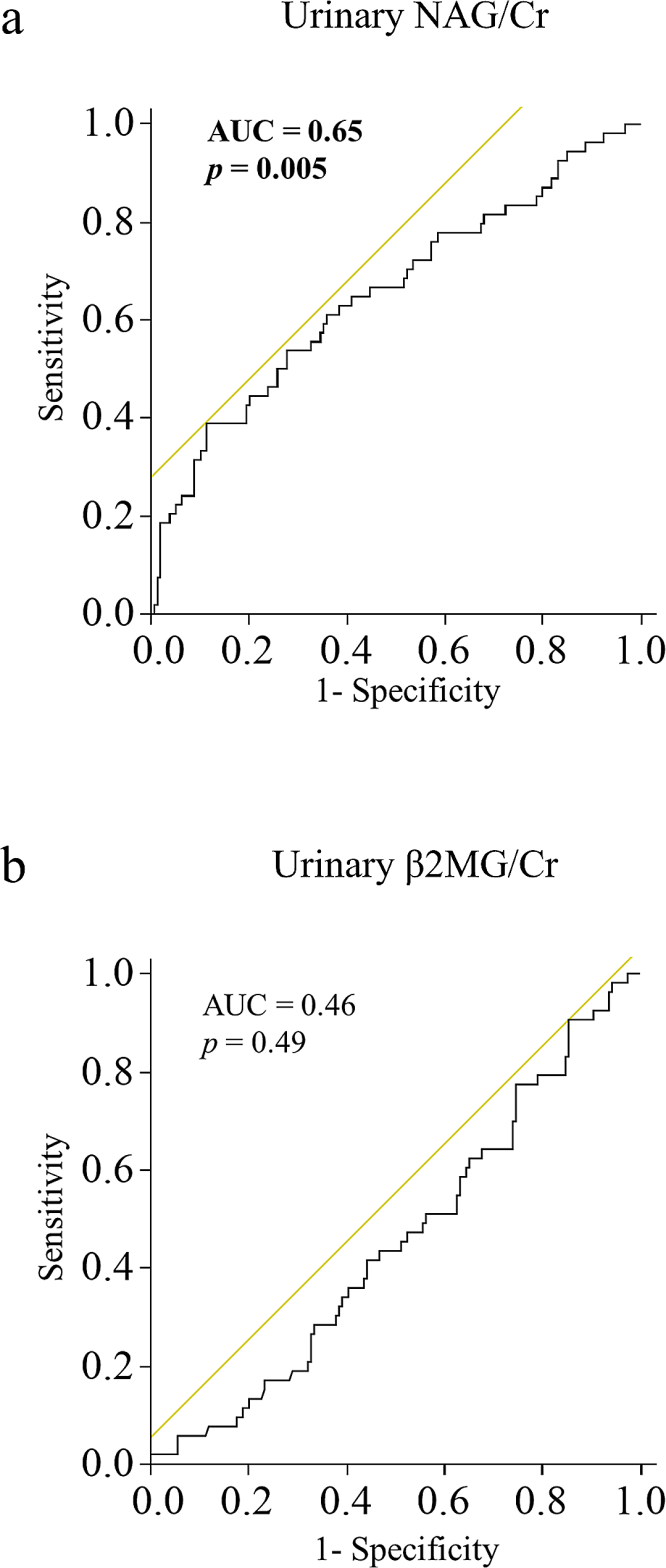
Receiver operating characteristics (ROC) curves. (**a**) Urinary N-acetyl-β-D-glucosaminidase (NAG)/Cr, (**b**) urinary β2-microgrobulin (β2MG). AUC: area under the receiver operating characteristic curve

Next, we investigated the predictive ability of the urinary NAG/Cr ratio for the incidence of Hb loss ≥ 1 g/dL due to a PKB using an ROC curve. The ROC curve showed that the urinary NAG/Cr ratio could predict the incidence of Hb loss ≥ 1 g/dL, with an AUC of 0.65 (*p* = 0.005, Fig. 3a). In contrast, the urinary β2MG level did not predict the incidence of Hb loss ≥ 1 g/dL (AUC 0.46, *p* = 0.49; Fig. 3b). The optimal cutoff value of the urinary NAG/Cr ratio for predicting the incidence of Hb loss ≥ 1 g/dL was 35 U/L.

We further examined whether a high urinary NAG/Cr ratio was a risk factor for the incidence of Hb loss ≥ 1 g/dL. In this study, we defined a high urinary NAG/Cr ratio as ≥ 35 U/L. The incidence of Hb loss ≥ 1 g/dL was higher in patients with dyslipidemia (odds ratio [OR] 1.95, 95% confidence interval [CI] 1.02–3.77, *p* = 0.047), abnormal APTT (OR 3.93, 95% CI 1.71–9.03, *p* = 0.001), urinary protein ≥ 3 g/gCr (OR 2.61, 95% CI 1.38–4.94, *p* = 0.003), and a high urinary NAG/Cr (OR 4.69, 95% CI 2.27–9.70, *p* < 0.0001; Table 3) than in others.

**Table 3:** Unadjusted and adjusted risks of post-biopsy hemoglobin loss ≥ 1 g/dL

Variables	*N*	Incidence, *N* (%)	Incidence, *N* (%)		uOR (95%CI)	*p* value		aOR (95%CI)	*p* value
		(positive)	(negative)						
Sex (male)	110	31 (28.2)	23 (22.3)		1.36 (0.73–2.54)	0.33		-	-
Age > 70	55	19 (34.6)	35 (22.2)		1.85 (0.95–3.63)	0.07		-	-
BMI without normal range	67	15 (22.4)	39 (26.7)		0.79 (0.40–1.56)	0.50		-	-
Hypertension	101	28 (27.7)	26 (23.2)		1.27 (0.68–2.36)	0.45		-	-
Diabetes	28	11 (39.3)	43 (23.2)		2.14 (0.93–4.91)	0.08		-	-
Heart disease	16	7 (43.8)	47 (23.9)		1.75 (0.94–3.26)	0.10		-	-
Dyslipidemia	60	21 (35.0)	33 (21.6)		**1.95** **(1.02–3.77)**	**0.047**		1.47 (0.71–3.04)	0.30
eGFR < 45 mL/min/1.73m^2^	87	25 (28.7)	29 (23.0)		1.35 (0.72–2.51)	0.35		-	-
Hb < 10 g/dL before biopsy	26	4 (15.4)	50 (22.8)		0.63 (0.20–1.94)	0.40		-	-
APTT without reference range	27	14 (51.9)	40 (21.5)		**3.93** **(1.71–9.03)**	**0.001**		**3.15** **(1.30–7.64)**	**0.01**
uPro/uCr > 3 g/gCr	71	27 (38.0)	27 (19.0)		**2.61** **(1.38–4.94)**	**0.003**		1.52 (0.72–3.18)	0.27
uNAG/uCr > 35 U/gCr	40	21 (52.5)	33 (19.1)		**4.69** **(2.27–9.70)**	**< 0.0001**		**3.21** **(1.42–7.27)**	**0.005**
Needle passes > 4 times	30	8 (2667)	46 (25.14)		1.08 (0.45–2.60)	0.86			
Acute kidney injury	13	5 (38.5)	49 (24.5)		1.93 (0.60–6.16)	0.28			
Vasculitis	18	6 (33.3)	48 (24.6)		1.53 (0.55–4.30)	0.43			

Data were analyzed using chi-squared test for the unadjusted OR, and statistically significant variables were further analyzed using a multiple logistic regression analysis for the adjusted OR. Akaike’s information criterion (AIC) for this analysis was 224.89 (*p* < 0.0001)

OR, odds ratio; CI, confidence interval; eGFR, estimated glomerular filtration rate; Hb, hemoglobin; APTT, activated partial thromboplastin time; uPro, urinary protein; Cr, creatinine; uNAG, urinary N-acetyl-β-d-glucosaminidase

A multivariable logistic regression analysis showed that abnormal APTT (OR 3.15, 95% CI 1.42–7.27, *p* = 0.005) and a high urinary NAG/Cr ratio (OR 3.21, 95% CI 1.42–7.27, *p* = 0.005) were independent risk factors for the incidence of Hb loss ≥ 1 g/dL (shown in Table 3).

In addition, another multivariable logistic regression model that used previously reported risk factors also indicated that a high urinary NAG/Cr was an independent risk factor for the incidence of Hb loss ≥ 1 g/dL (OR 5.32, 95%CI 2.46–11.5, *p* < 0.0001) (Table 4).

**Table 4:** Multivariate logistic regression analysis of factors associated with the occurrence of post-biopsy hemoglobin loss ≥ 1 g/dL, with adjustment for previously reported risk factors

Variables	OR	95% CI	*p* value
uNAG/uCr > 35 U/gCr	**5.32**	**2.46–11.5**	**< 0.0001**
Hb < 10 g/dL before biopsy	0.35	0.09–1.29	0.12
Vasculitis	1.38	0.42–4.58	0.59
Acute kidney injury	2.26	0.68–7.56	0.18

Data were analyzed using a multiple logistic regression analysis. Akaike’s information criterion (AIC) for this analysis was 224.313 (*p* < 0.0001)

OR, odds ratio; CI, confidence interval; eGFR, estimated glomerular filtration rate; Hb, hemoglobin; Cr, creatinine; uNAG, urinary N-acetyl-β-d-glucosaminidase

### Association between urinary NAG and histopathological findings in kidneys

**Fig. 4:**
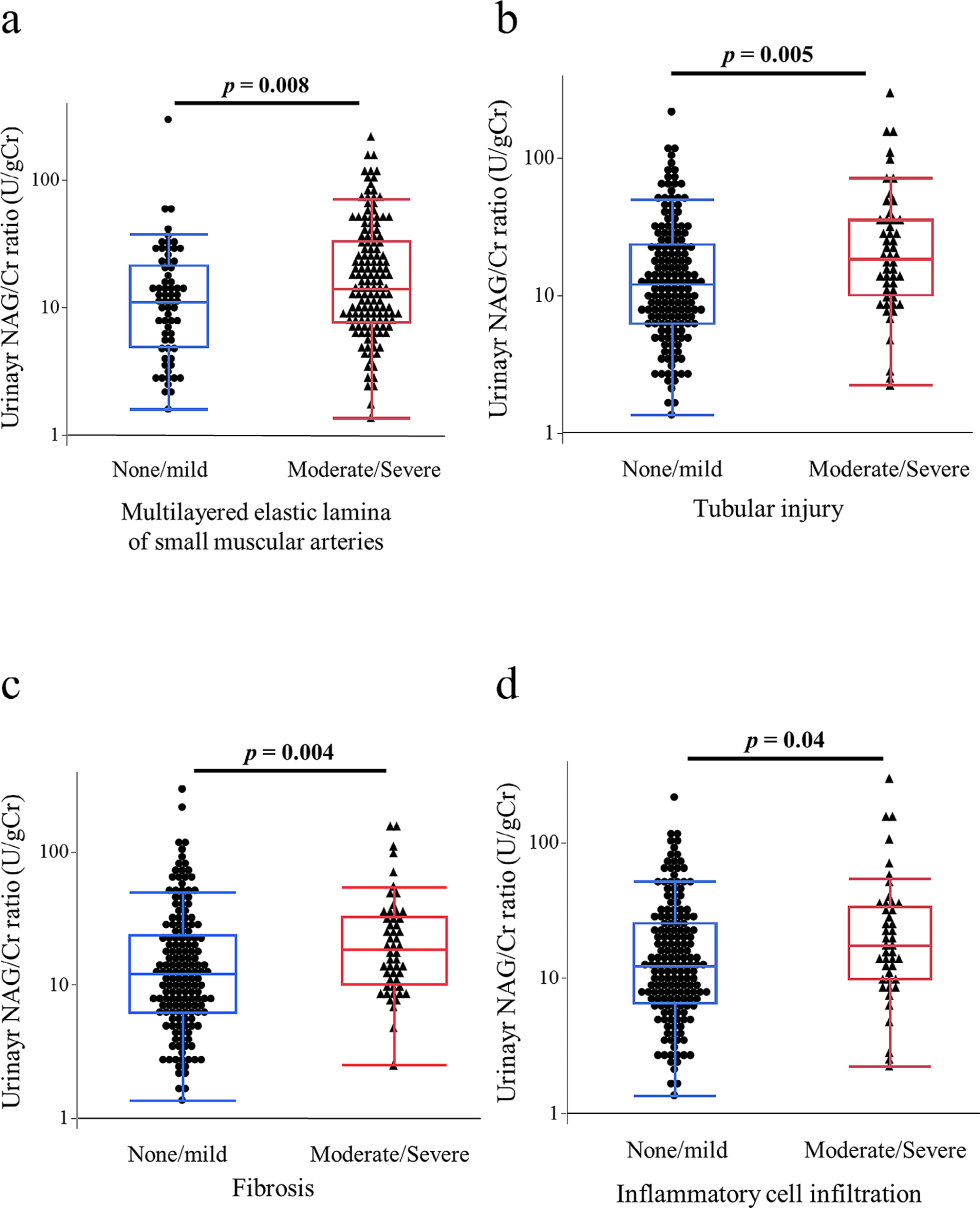
Comparisons of urinary NAG/Cr ratios by the severity of pathological findings. (**a**) Multilayered elastic lamina of small muscular artery, (**B**) tubular injury, (**c**) fibrosis, and (**d**) inflammatory cell infiltration. Patients were divided into two severity groups (none/mild vs. moderate/severe) for each pathological finding. Dots represent each individual. Box-and-whisker plots: within each box, horizontal lines denote the median values; boxes extend from the 25th to the 75th percentile of each group’s distribution of values; vertical extending lines denote adjacent values (i.e. the most extreme values within the 1.5 interquartile range [IQR] of the 25th and 75th percentile of each group). Data were analyzed using the Mann-Whitney U-test. NAG, N-acetyl-β-D-glucosaminidase

To explore why a high urinary NAG/Cr ratio was a risk factor for Hb loss ≥ 1 g/dL, we additionally evaluated the pathological findings of PKBs. The associations between the pathological findings and urinary NAG/Cr values are shown in Fig. 4. The urinary NAG/Cr values were significantly higher in patients with moderate-to-severe multilayered elastic laminae of the small muscular artery (*p* = 0.008, Fig. 4a), moderate-to-severe tubular injury (*p* = 0.005, Fig. 4b), moderate-to-severe fibrosis (*p* = 0.004, Fig. 4c), and infiltration of inflammatory cells (*p* = 0.04, Fig. 4d) than in those who had none or only mild pathological disorders.

**Fig. 5:**
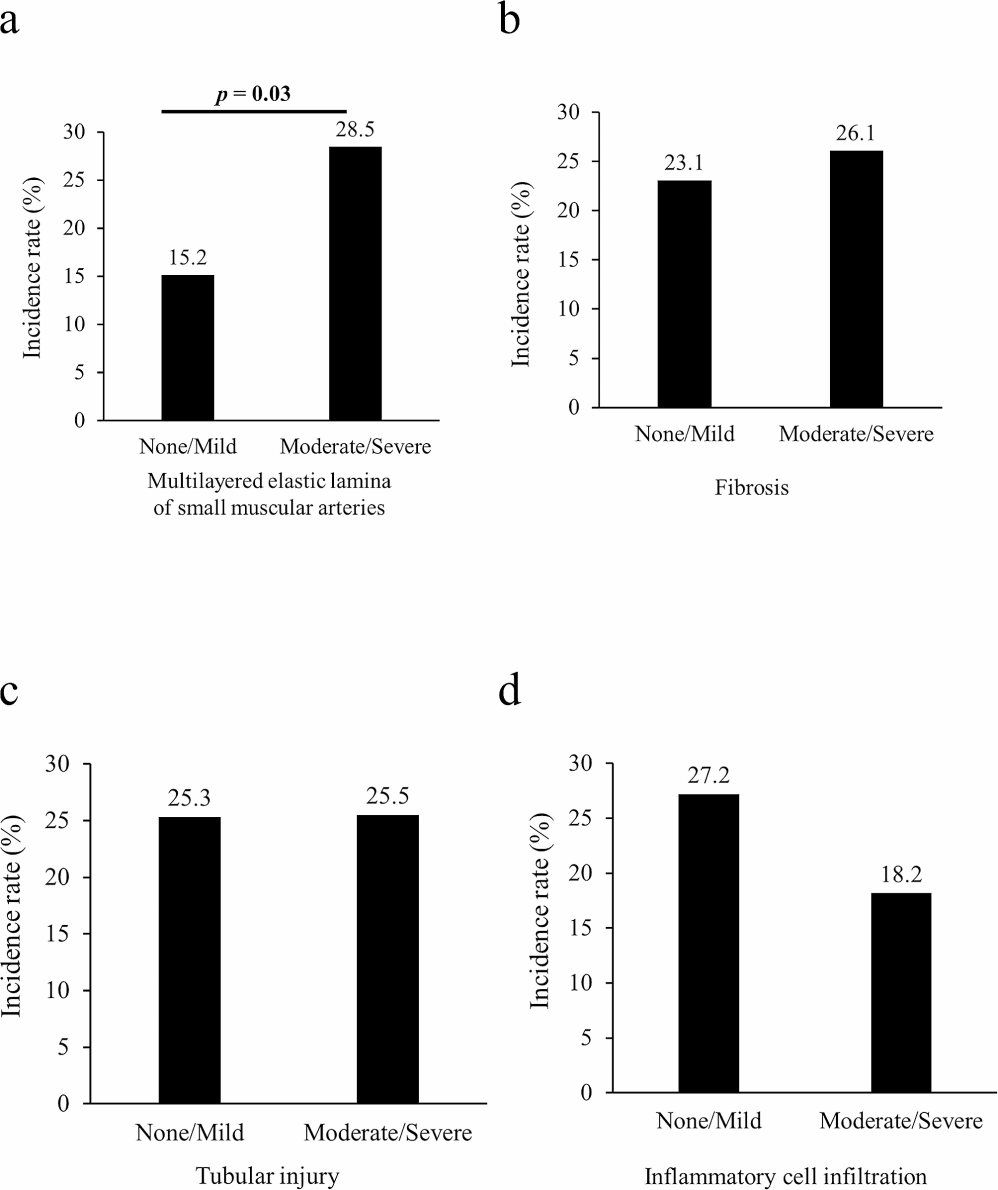
Comparisons of the incidence rate of hemoglobin (Hb) loss ≥ 1 g/dL by severity of pathological findings. (**a**) Multilayered elastic lamina of small muscular artery, (**b**) tubular injury, (**c**) fibrosis, and (**d**) inflammatory cell infiltration. Patients were divided into two severity groups (none/mild vs. moderate/severe) for each pathological finding. The numbers on the top of the bar graph represent the incidence rate as a percentage. Data were analyzed using Fisher’s exact test

The associations between pathological findings and the incidence rates of Hb loss ≥ 1 g/dL are shown in Fig. 5. The incidence rates of Hb loss ≥ 1 g/dL were significantly higher in patients who had moderate-to-severe multilayered elastic laminae of the small muscular artery than in those who had none or mild one (28.5% vs. 15.2%, *p* = 0.03, Fig. 5a). However, the severity of other pathological findings, such as fibrosis, tubular injury, and inflammatory cell infiltration, were not associated with an elevated bleeding risk.

## Discussion

The principal finding of this study was that urinary NAG could predict the incidence of Hb loss ≥ 1 g/dL after a PKB. Our result may suggest that particular attention should be paid when performing a renal biopsy in patients with high urinary NAG levels, and close follow-up after a biopsy should be performed in these individuals.

Urinary NAG measurement is now widely used in clinical practice. It is because the level of urinary NAG has been reported to be associated with the severity of various kidney diseases. A recent study reported that high urinary NAG levels in patients with IgA nephropathy were associated with an increased risk of disease progression [21]. In nephrotic syndrome, urinary NAG levels are higher in patients with steroid-resistant nephrotic syndrome than in those with steroid-sensitive nephrotic syndrome [14]. Another reason is that the measurement assay is rapidly quantified, highly reproducible, and well-defined. Considering our results, the measurement of urinary NAG prior to PKB may serve as an informative, rapidly applicable metric to help nephrologists understand the status of kidney disease and the risk of bleeding due to PKB.

In the present study, we showed that only the severity of multilayered elastic laminae of the small muscular artery were associated with both elevated urinary NAG levels and the incidence of Hb loss ≥ 1 g/dL. The severity of other pathological findings, such as fibrosis, tubular injury, and inflammatory cell infiltration, were not associated with an elevated bleeding risk, although these findings were all related to elevated urinary NAG/Cr levels. Urinary NAG levels appear to be related to arteriosclerosis, and some studies have shown that urinary NAG is related to microangiopathy in diabetic kidney disease [22, 23]. In addition, in patients with peripheral artery disease, high urinary NAG levels are associated with the risk of major adverse cardiovascular and cerebrovascular events [24]. Furthermore, arteriosclerosis may be associated with increased bleeding events after a PKB. A recent study showed that systemic arterial stiffness quantified by brachial-ankle pulse wave velocity was an independent risk factor for anemia after a PKB [25]. Increased pulsatile stress may damage small arteries and tear their endothelial and smooth muscle cells with disruption of the vessel [26], which can induce a hemorrhagic tendency. Therefore, our results suggest that atherosclerosis in the kidney may be the underlying mechanism by which urinary NAG predicts bleeding risk after a biopsy.

Although some studies have previously defined bleeding events as hemoglobin loss of ≥ 2 g/dL [7], we defined bleeding events as Hb loss of ≥ 1 g/dL in our study. This is because even a mild complication should be investigated to further enhance the safety of PKB. Furthermore, Hb loss of ≥ 1 g/dL implies a substantial amount of blood loss. For a person with a body weight of 80 kg, a 1 g/dL decrease in hemoglobin is calculated to be approximately equivalent to the loss due to donating a single unit (400mL) of whole blood. Chikamatsu et al. previously studied the association between Hb loss and bleeding volume after a PKB, quantified by CT [10]. They found that patients in the high-bleeding-volume group, whose mean estimated bleeding volume by computed tomography was 126 mL (25-75th percentile: 85–203 mL), experienced greater changes in Hb (mean ± standard deviation: -0.9 ± 1.0) after a PKB than patients with less bleeding. Therefore, an Hb decrease of only 1 g/dL after PKB seems to reflect a high bleeding volume. Some institutes do not routinely perform ultrasound examinations [2], and Hb loss of ≥ 1 g/dL would be an indicator for the next step of testing to investigate blood loss (e.g., ultrasound or CT).

Several limitations associated with the present study warrant mention. First, it was a single-center retrospective study. One study reported that a small clinical center size (< 30 PRBs/year) was associated with an increased risk of bleeding complications after a PKB [27]. Further studies should be conducted across heterogeneous clinical centers. Second, some factors that have been reported to be associated with bleeding events were not identified as independent risk factors in this study. These risk factors might be underestimated because patients with previously known bleeding risk factors are treated particularly carefully when undergoing a PKB. Therefore, prospective studies with robust protocols are needed to confirm the predictive ability of urinary NAG levels for bleeding events. Third, the sample size was relatively small because we limited the number of enrolled patients to within the past five years. However, with advances in biopsy techniques and equipment, the incidence of bleeding events has improved, and data from earlier PKBs may not reflect the current bleeding risk. Fourth, the incidence rate of bleeding events in this study may have been affected by Hb variability, which is influenced by age, sex, ethnicity, smoking status, and physical activity. Diurnal and seasonal variation in Hb have also been reported [28].

In conclusion, we demonstrated for the first time that a high urinary NAG level was associated with bleeding events after a PKB. Arteriosclerosis in the kidneys may be the mechanism underlying these increased bleeding events. Evaluating urinary NAG levels before a PKB may help predict not only the severity of kidney diseases but also the risk of bleeding events after a biopsy. A multicenter prospective study is needed to confirm the predictive ability of urinary NAG levels for bleeding events due to PKBs.

### Electronic supplementary material

Below is the link to the electronic supplementary material.

Supplementary Material 1

## Data Availability

Data will be made available upon request directed to the corresponding author. Proposals will be reviewed and approved by the investigators and collaborators based on scientific merit. After approval of a proposal, data will be shared through a secure online platform after signing the data access and confidentiality agreement.
