# Supplementary material for: Urinary N-acetyl-D-glucosaminidase can predict bleeding after a percutaneous kidney biopsy
Source: BMC Nephrol. 2024 Jul 22;25:234. doi: 10.1186/s12882-024-03658-z (PMC11265090; doi:10.1186/s12882-024-03658-z)
Supplement: Supplementary file 1 — Supplementary Material 1 [file 12882_2024_3658_MOESM1_ESM.docx]

**Supplemental Table of Contents**

This article contains the following supplemental table.

Supplemental Table. Indications and contraindications of PKB at our institute.

**Supplemental Table 1. Indications and contraindications of PKB at our institute**

| **Indication** | **Contraindication** |
| --- | --- |
| 1. **Hematuria with proteinuria and/or presumed renal origin** | **(1) Urinary tract infection** |
| 1. **Proteinuria (≥ 0.5 g/day or g/gCr)** | **(2) Uncooperative patient** |
| 1. **Suspected RPGN (hematuria with rapid decline of kidney function)** | **(3) Renal anatomic anomalies**  **(solitary native kidney/horseshoe kidney**  **/aneurism/polycystic kidney/** **hydronephrosis)** |
| 1. **Unexplained renal impairment** | **(4) Uncontrolled severe hypertension** |
| 1. **Renal involvement of systemic disease** | **(5) Uncontrolled bleeding diathesis/** **Antithrombotic therapy cannot be discontinued** |

PKB, percutaneous kidney biopsy; RPGN, rapidly progressive glomerulonephritis.
